# Estimating the effect of “treatment in the treated” - instrumental variable analysis vs conventional regression methods in the titre-2 trial in cardiac surgery

**DOI:** 10.1186/1745-6215-16-S2-P147

**Published:** 2015-11-16

**Authors:** Chris Rogers, Katie Pike, Jonathan Sterne, Barney Reeves

**Affiliations:** 1 University of Bristol, Bristol, UK

## Background

Perioperative anaemia is associated with adverse outcomes after cardiac surgery but, paradoxically, observational analyses have shown that red cell transfusion is associated with worse clinical outcomes. TITRe2 tested the hypothesis that a restrictive threshold for transfusion would reduce post-operative morbidity compared to a liberal threshold.

## Methods

Adults undergoing cardiac surgery with post-operative haemoglobin <9g/dL were recruited. Participants were randomised to transfusion if haemoglobin <7.5g/dL (restrictive) or <9g/dL (liberal). The primary analyses were by intention-to-treat. A secondary analysis of a composite outcome (serious infection or ischaemic event or death in the 3-months after randomisation) to assess the effect of receiving a transfusion was pre-specified. Two methods for handling confounding were applied: adjustment conventionally for covariates (CA) or using randomised allocation as an instrumental variable (IV).

## Results

2003 patients were randomised (1000 restrictive group, 1003 liberal group. Transfusion rates were 53.4% and 92.2% in the restrictive and liberal groups, respectively. The primary intention-to-treat analysis suggested a similar outcome in the two groups (odds ratio=1.11, 95%CI 0.91-1.34, p=0.30). In the CA analysis the odds of morbidity/mortality increased with transfusion (odds ratio=1.28 95%CI 1.03 to 1.60, p=0.028), but the IV analysis was in the opposite direction (relative risk=0.78, 95%CI 0.53-1.14, p=0.20).

## Discussion

CA analysis supports previous observational analyses and contradicting the primary analysis. IV analysis suggested a marginally protective effect of RBC transfusion consistent with the ITT analysis of the RCT. We conclude that the CA results are explained by residual confounding.

